# Exploring domains and ways of expressing gratitude in older couples: A qualitative study

**DOI:** 10.1177/20551029251406030

**Published:** 2025-12-03

**Authors:** Katharina Weitkamp, Michelle Roth, Michaela Baumann, Guy Bodenmann

**Affiliations:** 1 Department of Psychology, Clinical Psychology for Children/Adolescents and Couples/Families, 27217 University of Zurich, Zurich, Switzerland

**Keywords:** close relationships, gratitude, qualitative interview study, older couples, felt gratitude, expressed gratitude

## Abstract

In romantic relationships, gratitude promotes positivity, satisfaction and maintenance behaviour. However, little is known about specific domains of and ways of expressing gratitude in long-term relationships, particularly, as studies so far relied on pre-formulated self-report questionnaires that may fail to capture expressions of gratitude beyond direct verbal expressions. The research questions were: How do individuals experience gratitude in their current romantic relationship and how do they describe their partner’s expressions of gratitude? Forty-three older couples were interviewed separately by phone. Interview data were analysed with qualitative content analysis. Overall, a strong sense of gratitude was mentioned for the partner’s personality, the relationship quality, the partner’s support, their joint achievements and the life they have created together. Gratitude was expressed verbally, but also in idiosyncratic ways through love expressions, signs of affection, or gifts. To conclude, gratitude seems to be an integral part of long-term relationships and may be beneficial for healthy aging.

## Introduction

Although it is a common social phenomenon, gratitude has only recently received increased attention in research on couples and romantic relationships. Depending on the conceptualization, gratitude is understood either as a generally positive emotional state, a durable affective trait, a moral experience, or a character strength (Yoshimura and Berzins, 2017). Interpersonally, gratitude was defined by McCullough et al. (2008), as “a positive emotion that typically flows from the perception that one has benefited from the costly, intentional, voluntary action of another person” (p. 281). In this sense, gratitude may be understood as a benefit detector, reinforcer, and motivator of prosocial behaviour (McCullough et al., 2008).

Algoe and Zhaoyang (2016) observed the particular role of *responsive* action on the part of a benefactor and focused on the role of gratitude in close relationships. In their find-remind-bind theory, they state that gratitude is a detection-and-response system to help find, remind, and bind ourselves to attentive others (Algoe, 2012; Algoe et al., 2008). A further conceptualization of interpersonal gratitude differentiates between felt and expressed gratitude (Gordon et al., 2011). Thereby, felt gratitude describes a person’s experience of feeling grateful oneself whereas expressed gratitude describes a person’s tendency to share gratitude by expressing it (Gordon et al., 2011).

Both, generalized gratitude and gratitude within interpersonal relationships were shown to be beneficial. Interpersonally, gratitude seems to be beneficial for both senders and receivers individually (Algoe et al., 2008; Chang et al., 2022b; Lambert et al., 2010). Additionally, gratitude promotes positivity, satisfaction, and maintenance behaviour on the relational level (Gordon et al., 2011; Lambert and Fincham, 2011). Furthermore, beneficial effects of gratitude on relationship satisfaction have been widely documented (Algoe et al., 2008; Park et al., 2019). One person’s experienced emotion fuels an upward spiral of relational growth for each member of the dyad (Algoe and Zhaoyang, 2016), thereby pointing to the relevance of gratitude in intimate relationships in our daily lives (Algoe, 2012). Expressed gratitude also seems to be able to moderate the negative impact of financial strain on relationship quality, mediated by destructive communication patterns (i.e., demand-withdraw), and therefore plays a key role in understanding positive buffering effects (Barton et al., 2015). Additionally, perceived or felt gratitude was more important than expressed gratitude in a study by Gordon et al. (2011). In terms of gender roles, some differences between men and women may exist regarding the experience and expression of gratitude. Men compared with women were less likely to feel and express gratitude, men made more critical evaluations of gratitude, and derived fewer benefits from it (Kashdan et al., 2009).

Even though numerous studies found beneficial effects of gratitude, little is known about specific sources of gratitude within romantic relationships. Gratitude, conceptualized as a positive emotion, can vary in intensity, much like any other affective state (Brehm, 1999). The source of gratitude may significantly influence its magnitude; for instance, a minor favor extended by a partner might evoke only a modest sense of gratitude, whereas profound support during times of acute need is likely to elicit a deeper emotional response (Roth et al., 2024). This in turn is believed to have stronger positive effects for both oneself and the partner (Roth et al., 2024). Given the relevance of gratitude for both individuals and couples as outlined above, knowledge about domains of gratitude might be used to design effective gratitude interventions for couples.

Additionally, it may be assumed that gratitude expressions are not merely verbal expressions of thankfulness without any nonverbal cues or context. Rather, these expressions likely vary along verbal and nonverbal channels with a number of potential meanings for both senders and receivers (Yoshimura and Berzins, 2017). However, little is known about different ways of expressing and perceiving gratitude in romantic couples. To date, as Yoshimura and Berzins (2017) state in their conceptual paper on gratitude, we know little about what kind of messages are understood distinctly as one of gratitude rather than a message of affection, compassion, politeness, kindness, or other constructs. This lack of knowledge may be partly due to the majority of studies on gratitude relying on pre-formulated items in self-report questionnaires. This allows no insight into the subtleties and complexities of gratitude exchanges in intimate relationships. Thus, as a relevant next step, the qualities of gratitude expressions need to be described in more detail to generate more accurate theoretical models (Yoshimura and Berzins, 2017) as well as to derive practical implications for preventive couple programs and couple therapy.

When studying expressions of gratitude in intimate relationships, it appears valuable to focus on older couples who have been in long-term relationships (couples who are in committed relationships for at least 10 years). While other processes such as infatuation, sexual attraction, or idealization dominate in the early phases of the relationship, gratitude could take on a stabilizing function in longer partnerships - for example by promoting prosocial behaviour, facilitating forgiveness or strengthening long-term reciprocity (e.g., Algoe, 2012). Long-standing relationships are at risk of being taken for granted (“It’s normal for you to do that”), which can lead to a decline in perceived dyadic coping and mutual appreciation. Therefore, gratitude can represent a counter-movement to the devaluation of the everyday in long-term relationships (Kubacka et al., 2011). Furthermore, couples who have been together for 10 years or more are more likely to have experienced life transitions (e.g., transition to parenthood) and major stressors, and therefore might feel more gratitude towards the partner (Gordon et al., 2012).

Especially since gratitude seems to be higher in older adults compared with younger adults (Chopik et al., 2019) and recent work showed that dispositional gratitude matters for their life satisfaction (Chang et al., 2022a) studying the phase of late adulthood may be worthwhile. In late adulthood, according to Erikson (1982) the developmental stage of Ego Integrity versus Despair, individuals are taking stock, looking back at their lives. If this looking back on the life lived is done in a positive way, it seems closely connected to the experience of gratitude (McAdams and Bauer, 2004). Some evidence points to the notion that different subjective experiences of life may be due to a limited future time perspective rather than chronological age (Allemand and Hill, 2016). Additionally, with the ageing societies across the globe, supporting healthy ageing becomes a public health imperative (Pruchno, 2021). Promoting gratitude could be a low threshold avenue to increase mental well-being in old age (Tani et al., 2022), potentially designing interventions on the couple level as well (Parnell et al., 2020). Thus, the current study aims to examine gratitude exchange processes in long-term relationships in older couples using qualitative interview data.

### Research questions

Concepts from positive psychology have received increased attention in health psychology over the past years highlighting their relevance for physical health outcomes (Aspinwall and Tedeschi, 2010; Rasmussen et al., 2009). While positive effects of gratitude in romantic relationships are well-documented (Algoe and Zhaoyang, 2016), the context in which gratitude may arise and ways in which gratitude is expressed are so far neglected. Also, gratitude may be a relevant protective factor within health psychology particularly for older adults, as this population often faces increased physical health challenges that can threaten overall well-being. Conversely, little is known whether experiences of ingratitude play a role in long-term relationships. It is also important to distinguish between felt gratitude and received gratitude. Understanding these nuances can inform the development and refinement of targeted interventions aimed at enhancing gratitude and mitigating its absence, thereby promoting better health outcomes and psychological well-being in aging populations. Based on the lack of research on subjective experience and expression of gratitude within long-term relationships, the following research questions formed the basis of the qualitative data analyses: (1) How do individuals experience gratitude in their current romantic relationship? (2) How do spouses describe their way of expressing gratitude towards their partner? (3) How do spouses describe their partner’s expressions of gratitude?

## Methods

### Recruitment and participants

We were interested in couples who were in a long-term relationship, which we defined as being in a committed relationship for at least 10 years regardless of their marital status. The current data was collected as part of a larger longitudinal study following romantic couples across a time span of 10 years (Kirkhoff et al.). The study consisted of 10 annual measurement time points. Couples were initially recruited in 2011 via radio and newspaper advertisements. To be eligible for initial study participation, couples had to be in their current relationship for at least 1 year, over 18 years of age, and having sufficient command of the German language. In total, 368 mixed-gender couples were included at first assessment. Participating couples were grouped into three age cohorts: age-group 1 ranged from 20 to 35 years (*n* = 122 couples), age-group 2 ranged from 40 to 55 years (*n* = 125), and age-group 3 ranged from 65 to 80 years (*n* = 121). Over the course of the study, questionnaire data, behavioural observation data, and qualitative interview data were collected.

The current study draws on data from the last measurement time point when the qualitative interviews were conducted and focuses on the oldest age cohort only. The final sample consisted of *N* = 43 mixed-gender couples. Women were on average *M* = 77.40 (*SD* = 4.12) and men *M* = 79.22 (*SD* = 5.37) years old. Relationship duration was on average *M* = 49.97 years (SD = 15.23). Eighty-nine percent of the couples were married (some in their second marriage). The rest of the couples was not married to each other, with some being divorced from former partners; one woman was widowed from a former partner. All but one couple lived together. The sample showed a high level of education (see Table 1).

**Table 1.** Sample characteristics for women and men separately.

Variable	Women (*n* = 43)		Men (*n* = 43)	
	M	SD	M	SD
Age in years	77.40	4.12	79.22	5.37
Relationship duration in years	49.97	15.23	-	-

	*n*	%	*n*	%
Level of education				
Mandatory school	2	5	0	0
Vocational training	18	42	13	30
High school	12	28	5	12
College/university	11	26	25	58
Living situation				
Together with partner	42	98	40	93
Alone	0	0	1	2
Shared living (with partner)	0	0	2	5
Shared living (without partner)	1	2	0	0
Civil status				
Married (1st marriage)	33	77	33	77
Married (2nd marriage)	5	12	5	12
Unmarried	-	-	1	2
Divorced	4	9	4	9
Widowed	1	2	-	-
Children/Step children (yes)	39	91	37	86

### Interview procedure

All procedures were evaluated by the Ethics Committee of the Philosophical Faculty of the University of Zurich (No. 2013.1.1, No. 17.8.2; No. 19.8.13; No. 20.6.18; No. 24.08.27). Participants were informed about the study and gave their written informed consent. Qualitative data from the analyses presented here were part of individual phone interviews (February – July 2021). Interviews were carried out by trained graduate students via phone with study participants individually. Interviews were conducted in Swiss German and were audio recorded and transcribed verbatim. Interview duration was on average *M* = 24.28 min (*SD* = 8.15; range 10 to 46 min). The semi-structured interview guideline covered dyadic coping – a concept, which views stress and coping as an interpersonal phenomenon, where stress signals of one partner are perceived, interpreted and answered by another partner (e.g., Systemic Transactional Model, STM; Bodenmann, 1997, 2005) – and gratitude within the relationship. In the current study, we focused on the qualitative data on gratitude within the relationship. Specifically, participants were asked about their experience of gratitude towards their partner, the expression of their feelings of gratitude towards their partner, and the the experience of the partner’s gratitude towards oneself.

### Qualitative analyses

Interviews were analysed following qualitative content analyses as laid out by Kuckartz and Rädiker (2022). The aim of this hermeneutic method is to identify and conceptualise the content-related aspects relevant to the research question using categories. In the first phase, coders familiarized themselves with the data, noted essential observations for each couple in memos and kept a logbook throughout the analyses process. The main categories were formed based on the research questions. Definitions for these main categories were recorded in code memos and formed part of the subsequent codebook. Kuckartz and Rädiker (2022) suggest that approximately 10%–25% of the analysed material should be test-coded in order to check the category system for applicability to the empirical material. In this case, interviews from 10 dyads (23% of the data material) were test-coded and changes were made throughout this process. The main categories were inductively formulated based on the material and divided into further subcategories where necessary. These subcategories were labelled and described using quotations from the material. The procedure was inductive with the exception of gratitude for received support and dyadic coping. Since the research focus of the main study was on dyadic coping, we were interested whether and how participants would touch on this topic.

To assess dependability, the first nine dyads (21% of the data material) were coded independently by two coders (KW, MB) on the basis of the codebook. Intercoder agreement was calculated in MAXQDA (VERBI Software, 2023). High intercoder reliability of κ_n_ = .89 was found for these double-coded interviews (Brennan and Prediger, 1981). Subsequently, sections with inconsistent codes were marked and discussed. Thus, the category definitions were clarified and categories with insufficient specificity were identified and amended. Next, the whole data set was coded by (MB) and uncertainties were resolved through discussion with the research team.

## Results

**Figure 1.**
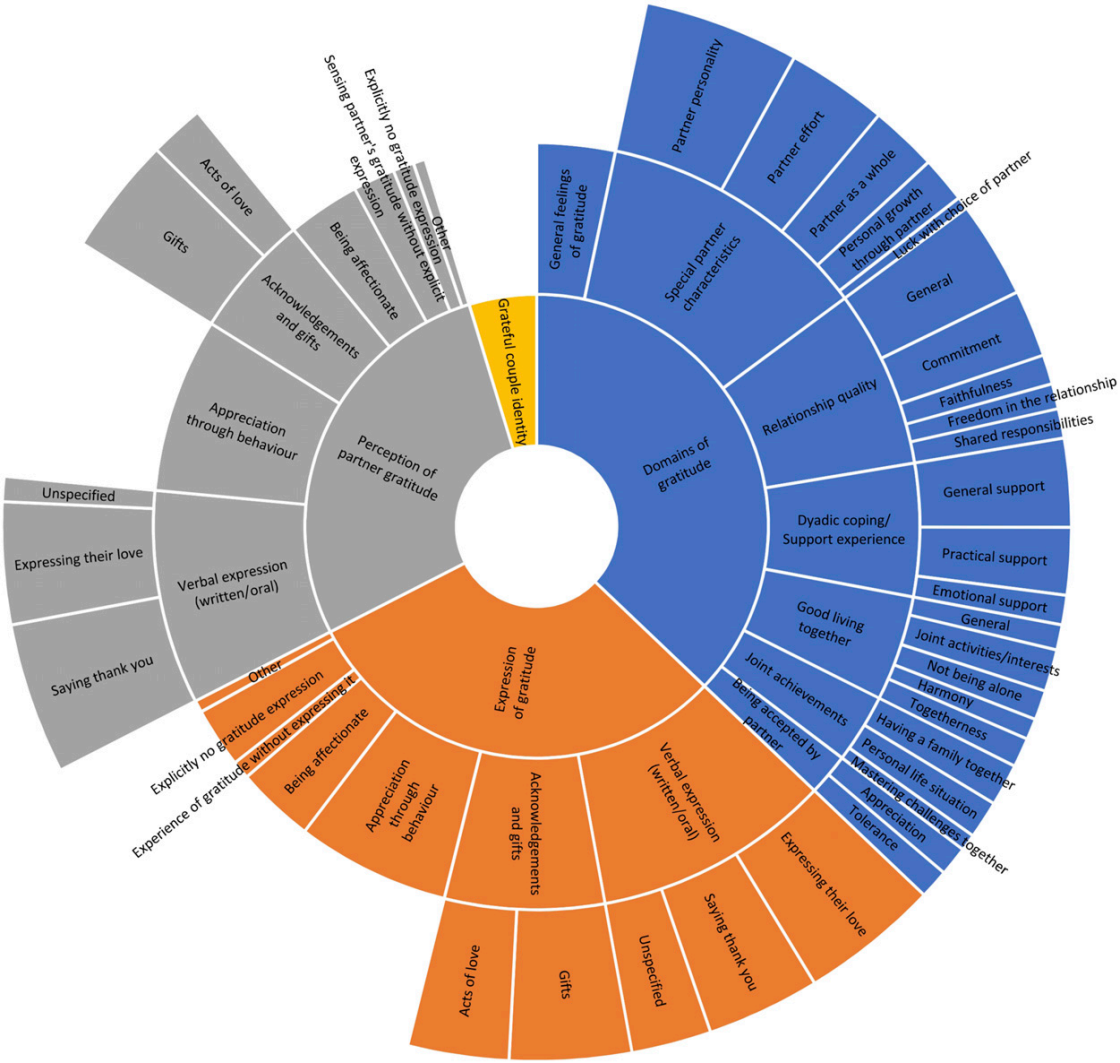
Absolute category distribution of reported facets of gratitude experience and expression.

The qualitative analyses led to four main categories covering the experience and expression of gratitude within long-term relationships of older couples: *domains of gratitude, expression of gratitude*, *perception of partner gratitude,* and *grateful couple identity.* For a detailed overview of the final category system with subcategories and frequencies see Table 2 and Figure 1. In the following, we present our findings based on the research questions, which are in-line with the main categories. The expression of one’s own gratitude and the experience of partner gratitude will be presented together, since there was considerable overlap. Due to space limitations, we report only those subcategories that were mentioned by more than 8% of the sample (total person frequency) in greater detail. Additionally, we mention rare findings when they conveyed something surprising or particularly meaningful.

**Table 2.** Main and subcategories of the coding system for experience and expression of gratitude in long-term relationships.

Main categories categories subcategories	Frequencies		
	Absolute topic frequency^a^	Absolute person frequency	Relative person frequency (%)^b^
Domains of gratitude			
General feelings of gratitude	26	25	20.9
Joint achievements			
Having a family together	10	10	9.3
Personal life situation	9	9	10.5
Mastering challenges together	4	4	4.7
Special partner characteristics			
Partner personality	37	30	34.9
Partner effort	24	23	26.7
Partner as a whole	16	15	17.4
Personal growth through partner	11	9	10.5
Luck with choice of partner	3	3	3.5
Relationship quality			
General	23	19	22.1
Commitment	16	14	16.3
Faithfulness	7	7	8.1
Freedom in the relationship	6	6	7.0
Shared responsibilities	7	5	5.8
Dyadic coping/Support experience			
General support	21	19	22.1
Practical support	16	15	17.4
Emotional support	7	6	7.0
Good living together			
General	6	6	7.0
Joint activities/interests	10	10	11.6
Not being alone	7	7	8.1
Harmony	5	5	5.8
Togetherness	7	5	5.8
Being accepted by partner			
Appreciation	7	7	8.1
Tolerance	7	7	8.1
Expression of gratitude			
Verbal expression (written/oral)			
Expressing their love	33	26	30.2
Saying thank you	27	26	30.2
Unspecified	19	19	22.1
Acknowledgements and gifts			
Gifts	29	28	32.6
Acts of love	24	21	24.4
Appreciation through behaviour	51	34	39.5
Being affectionate	26	24	27.9
Experience of gratitude without expressing it	6	6	7.0
Explicitly no gratitude expression	20	18	20.9
Other	4	4	4.7
Perception of partner gratitude			
Verbal expression (written/oral)			
Saying thank you	36	34	39.5
Expressing their love	29	24	27.9
Unspecified	6	6	7.0
Appreciation through behaviour	58	41	47.7
Acknowledgements and gifts			
Gifts	28	26	30.2
Acts of love	13	13	15.1
Being affectionate	24	20	23.3
Sensing partner’s gratitude without explicit expression	14	13	15.1
Explicitly no gratitude expression	7	7	8.1
Other	4	4	4.7
Grateful couple identity	37	23	26.7

Note. Codes with less than three mentions were omitted.

^a^Absolute frequency of coding for each category across all participants.

^b^*n*% of participants that have addressed the respective categories at least once.

### Domains of gratitude

Overall, we coded 390 data segments that touched on domains of gratitude in the interviews (see also Table 2). On a descriptive level, women expressed more domains of gratitude than men (216 coded segments compared with 174 coded segments).

#### General feelings of gratitude

A number of respondents (*n* = 25 people, 20.9%) reported that they felt a general sense of gratitude towards their partner. This was described as gratitude towards the partner for “everything” (dyad 3048, woman), “the whole package” (dyad 3078, man), or “many, many little things” (dyad, 3107, man). These accounts often permeated a deep sense of gratitude about their relationship. One interviewee described:

It’s just a trough of gratitude, isn’t it? It’s a heap of little things that, yeah, that kind of made me happy. (Dyad 3016, man)

#### Joint achievements

About a quarter of participants mentioned joint achievements as a source of gratitude (*n* = 21, 24.4%), such as building a life together, mastering demanding challenges like the death of a child (dyad 3078, man). Mostly, participants expressed gratitude for the family life that they have created together, the shared ups and downs of parenthood and from today’s perspective, gratitude for having created a large family with close family bonds. As one participant stated:

I am grateful that we have started a family and that we have built a good relationship with the children as well and that we have tended to this actually up until now. (Dyad 3023, man)

Another source of gratitude was the comfortable living situation, which included financial security, independence, the housing situation and the standard of living in general. Strikingly, it was predominantly women who mentioned this, for example, in the following statement:

…that he made a very nice life possible for me, in a nice flat with / we were able to bring up the children well, we were always going on crazy holidays. (Dyad 3049, woman)

#### Special partner characteristics

More than half of participants (*n* = 53, 61.6%) mentioned specific characteristics of the partner for which they were grateful for. Some described a general sense of gratitude for their partner as a whole and the partner’s presence in their life. One participant described it in the following way:

I simply say that I am grateful that I have my wife. She’s a good woman. And I love her very much. (Dyad 3022, man)

The partner’s personality and their behaviour were often mentioned as a source of gratitude. In particular, being reliable, consistent, calm, or level-headed were characteristics appreciated by their partners. As one woman described:

I am very grateful for his thoughtful / but not in a negative sense / his thoughtful, calm approach to life. (Dyad 3016, woman)

Others mentioned their partner’s humour, spontaneity, and creativity or the social and communicative skills of the partner in dealing with themselves or their social environment. Additionally, a strong sense of family values or being kind and attentive was expressed as reasons for feeling gratitude towards their partner. Some participants, mainly women, emphasised the lack of negative characteristics, such as the partner not being unfaithful or not having a drinking problem as something they were grateful for.

Yes, that he was always good to me, that he didn’t have other women or drink or whatever else there might be. (Dyad 3018, woman)

Furthermore, gratitude was reported for the fact that their partner was a driving force in enabling personal growth, encouraging professional advancements, or adventurous activities.

For which I am grateful to him, through him I have become more self-confident in a way. In other words, I have learnt to defend myself a little. (Dyad 3106, woman)

Gratitude for their partner’s contribution to their life together was expressed several times. Among other things, participants were grateful for the partners commitment to everyday tasks such as running the household, being a pillar throughout their professional life, ascertaining financial security, raising their children, dealing with grandchildren, or maintaining social relationships were mentioned. Major life events were also mentioned, as the following example shows:

I’m grateful to her for taking on the birth of our son, for example. Because I know that it’s a very painful, stressful situation for a woman, so I’m very grateful to her. (Dyad 3113, man)

#### Relationship quality

Gratitude in relation to the quality of the partnership was expressed by *n* = 38 respondents (44.2%). Many people explained that they were generally very grateful for their close relationship, for the long time they managed to stay together through “ups and downs” (dyad 3049, woman). Experiencing trust in the relationship and their partner, the willingness to stay together even in challenging times, and the partner’s commitment were domains of gratitude as well. The relationship was often described as happy or loving and a source of joy and pride, which can lead to gratitude, as the following example shows:

...joy in the relationship that we have created. […] And that is already / already something great. So there / I think / I’m very grateful for that. (Dyad 3086, man)

For some, traditional roles in the relationship were a source of gratitude, while others – in particular women – were grateful for being able to escape traditional gender roles to a certain degree, like having a paid job in spite of child care tasks, having a more balanced division of labour or a change of roles since retirement. Men mentioned being grateful for their partners taking on the traditional role of caretaking of the children and the household.

#### Dyadic coping/support experience

Thirty-five participants (40.7%) expressed gratitude for partner’s dyadic coping they received in times of stress but also partner support with daily activities. In most cases, this support was described as a general feeling of being able to rely on each other when necessary:

Just knowing in difficult moments he’s there. And stands by me. And then we get through it together. (Dyad 3086, woman)

Those who specified the type of support often described it as practical support such as assistance with household chores, practical help, and advice. Emotional support was mentioned to a lesser degree but often in relation to challenging life experiences. An example for practical support:

Like when I’m not feeling well, that he goes shopping or that he helps me hang out the washing or that he helps me in the garden outside. Yes, yes, those are some things that I’m very grateful for. (Dyad 3078, woman)

#### Good living together

Twenty-two participants (25.6%) mentioned gratitude for the beautiful life they created together with their partner, loving the partner’s presence, physical tenderness, and shared activities and interests.

Bike rides. Yes, we go on nice trips. That’s great, of course, if you can do that together, so / Have lots of hobbies together or go to a concert / cinema. (Dyad 3059, man)

Some also mentioned gratitude for not being alone in their older age.

#### Being accepted by partner

Gratitude for feeling accepted by their partner was expressed by *n* = 13 people (15.1%). Of these, about half reported gratitude for being appreciated by their partner (such as getting involved with their ideas, recognizing one’s expertise in certain areas, or feeling that they are taken seriously and supported in their own development), while another half described gratitude for the tolerance of their own person, which is not always easy (to endure, accept and deal patiently with one’s own nature, challenging personal characteristics, or difficult periods in life). For example, one interviewee described

Yes, well, she has effectively managed to accept my sometimes-difficult nature and deal with it. That is / that is very / very important. (Dyad 3026, man).

On a general note, some participants commented on the central role of gratitude in itself in maintaining and cultivating a long-term relationship.

I just don’t take it for granted. We’ve now been married for 50/ yes, soon 51 years and it’s actually always gone well. [...] And gratitude, I think that’s an important part of a relationship, or that you/ that you value the other person and/or express that too. (Dyad 3053, woman)

Interestingly, not all individuals perceived gratitude as a positive part of close relationships. For one participant, gratitude appeared to be incompatible with a close relationship, feeling more like a “foreign element”. This individual viewed caring for their partner as a given, rather than something that necessitates gratitude.

I’m not sure if gratitude is the right word (laughs). I think gratitude... / I perceive gratitude as something I could have towards other people if there is a reason. But with my partner the word does not fit somehow. It is / I can perhaps say that I am happy, I am (...) very satisfied, I am / perhaps even feel privileged from time to time, when the support is there. But gratitude doesn’t seem to me to belong in a partnership. [...] Gratitude [is] like something / a foreign element […] in a very close relationship. (Dyad 3112, man)

### Expression of gratitude

Expression of own gratitude and perception of partner’s gratitude expressions will be reported together due to the considerable overlap of categories. All participants remarked on some form of their expression of gratitude within their relationship, and also commented on their experience of the partner’s expressions of gratitude (see Table 2). A small number of participants mentioned that they would feel gratitude but would not or have not expressed this towards their partner. Some of these reports contained a sense of regret:

I feel it [the gratitude] more for myself. And every now and then, in retrospect, I reproach myself a little for not expressing this gratitude, or this love and joy with him, enough. Yes. (Dyad 3008, woman)

Complementing this, *n* = 14 people (15.1%) stated they would sense that the partner was grateful without the partner explicitly having to show it. These were mostly women, and some of them showed an understanding for their partner’s reluctance to express gratitude, because their partner was emotionally more reserved:

Only he’s not like that/he’s rather reserved emotionally. But I can tell that he’s grateful for a lot of things. And he can’t express it so intensely. (Dyad 3033, woman)

#### Verbal expression (written/oral)

Participants reported they expressed their gratitude through words (*n* = 62, 72.1%) most often, or they perceived the partner used written or oral ways to express their gratitude towards them (*n* = 55, 64%). Here, participants mentioned explicitly thanking for concrete things or support received from the partner during their day-to-day interactions or in the evening when reflecting back on the day. Indirect forms of expressing gratitude were also quite often mentioned, like spontaneous expressions of love, compliments, love letters, or poems.

Also, sometimes at the end of a day or something, when you look back, that you really emphasise that again and say thank you, yes, just verbally. (Dyad 3055, man)

#### Acknowledgements and gifts

Expressing gratitude through small acknowledgements and little gifts was mentioned by *n* = 38 (44.2%) and experienced from their partner by *n* = 36 (41.9%). These acts of gratitude spanned a range of gifts and behaviours, from cooking a favourite dish, giving massages, bringing home flowers, paying extra care in decorating the house, or celebrating anniversaries. One woman described the following way in which she expresses her gratitude:

When I know that he is interested in a certain book but would not go and just buy it, then I might buy it and put it under his pillow. (Dyad 3037, woman)

Interestingly, some participants explicitly stated that giving flowers or presents was experienced as a form of bribery and did not feel adequate to express their gratitude towards their partner.

This [giving presents] does not happen with us. We do not bribe each other (laughs). (dyad 3029, woman)

#### Appreciation through behaviour

Showing appreciation in their interactions was viewed as an indirect way to express gratitude and used by *n* = 34 (39.5%), whereas *n* = 41 (47.7%) experienced their partner’s gratitude like this. Loving and respectful ways of interacting, feeling the partner is proud of oneself, recognition of the partner or having a caring attitude were mentioned.

He cooks and handles things and so on, also very caring. Caring is also gratitude. (Dyad 3002, woman)

Twenty-four people (27.9%) said they expressed gratitude to their partner through affection, while *n* = 20 people (23.3%) reported receiving gratitude in the form of affection from their partner. Many described affection in the form of touching, hugging, kissing, sexual interactions, or caressing, as one example describes here:

...maybe I hug her a little or give her a peck or something. (Dyad 3116, man)

#### Grateful couple identity

About a quarter of participants (*n* = 23) perceived gratitude as being an essential element of their relationship that is experienced together. One woman stated how their relationship and gratitude cannot be viewed separately:

That we are immensely grateful for our life together. And I don’t think you can really separate the two. So, for me now, for us, because we’re actually grateful for our love, we’re grateful for our life, for our health. So, it seems to me that the two go hand in hand. (Dyad 3022, woman)

Some of the couples had very idiosyncratic ways of expressing their gratitude, including the incorporation of shared rituals. Examples of these rituals include jointly expressing what they are both grateful for during daily breakfasts or reflecting back on their relationship at certain occasions.

Or when we go hiking together / we go hiking together a lot and so / so out in nature, […] I say that a lot, I am very grateful that I was allowed to go this way with him and maybe a little further. (Dyad 3016, woman)

## Discussion

In the current study, we attempted to enrich and extend the knowledge on the sources and ways of expression and perception of gratitude in long-term romantic relationships in older age. To this end, we analysed qualitative interview data from both partners of 43 couples. For many interviewees the experience of gratitude seemed to play an important role in their long-term relationship. This finding aligns with research that highlights the relevance of gratitude in romantic relationships and its various functions in forming and maintaining relationships with the people that we interact with on a daily basis (Algoe et al., 2013). However, the clear significance of gratitude in the interviewees’ relationships contrasts with the limited understanding of gratitude as simply an emotion of reciprocal altruism for forming new social ties. For instance, McCullough et al. (2008) suggested that gratitude toward kin is less intense than gratitude toward non-kin. However, we saw deep feelings of gratitude within the romantic relationship.

So far, gratitude has been assessed in a general way without paying much attention to the various domains of gratitude in romantic relationships (e.g., Chang et al., 2022b). One main finding of our qualitative content analysis was the variety of domains of gratitude mentioned by the couples, which indicates that gratitude in a romantic relationship is evoked by a variety of situations and behaviours and is strongly characterised by the individuals and their shared life story. Some of the domains of gratitude that were mentioned align with research findings on gratitude, for instance, that people are grateful when they feel that their needs are being met (Algoe et al., 2008; Kubacka et al., 2011) or for the partner’s investment in the relationship (Joel et al., 2013). Participants touched on several relationship aspects that were previously found to be beneficial factors for relationship satisfaction. For instance, commitment (Le and Agnew, 2003) or feeling accepted by their partner (Rossman et al., 2022). Another source of gratitude that participants mentioned was engaging in dyadic coping with their partner in times of stress. From the interviews, it was clear that dyadic coping, as well as support in daily chores, seemed to play an important role in experiencing gratitude towards their partner. Some of the participants were grateful for the partner´s support in helping them develop their personality, like being more assertive or broadening their horizons. This deeper understanding of the various domains of gratitude has important implications for intervention programs and for practitioners working with (older) couples. On one hand, these identified domains can serve as a valuable framework for couples who might struggle to recognize specific sources of gratitude. Therapists and health psychologists can also draw on these domains when guiding couples in exploring and expressing gratitude within the relationship. Furthermore, the recognition of distinct gratitude domains can inform the refinement of existing preventive interventions for couples. For example, the Couples Coping Enhancement Training (CCET; Bodenmann and Shantinath, 2004) an effective prevention program designed to strengthen relationship skills, could be enhanced by integrating components that promote mutual expression of gratitude.

Additionally, insights into the sources of gratitude may stimulate future research and theoretical development on gratitude. While many existing studies assess gratitude on a particular day, our findings indicate that several key domains – such as joint achievements – may not occur on a daily basis and thus might be overlooked in such surveys despite their potential importance for couple’s well-being. These considerations highlight the need to account for more overarching facets of gratitude experiences within romantic relationships when designing research studies and interpreting their results.

Also worth mentioning is the integral role that gratitude seemed to play for some couples who stressed that shared gratitude was part of their couple identity. Some couples shared specific rituals of expressing mutual gratitude. This finding relates to what Lambert et al. (2009) termed generalized gratitude, as a distinct orientation towards life. Future research could delve more deeply into the potential benefits of a shared grateful couple orientation towards life, akin to the grateful couple identity observed in our study sample.

Research suggests that people in later life would be particularly grateful (Chopik et al., 2019). This was mirrored in the couples’ detailed accounts of domains of gratitude in the relationship and was palpable throughout our interviews. Even though we did not directly compare the expressed gratitude with younger samples, who may be equally grateful. Nonetheless, many interviewees expressed gratitude about living for such a long time and the sheer length of time being together in their relationship. This is in line with the gratitude-enhancing perception of a limited lifetime horizon as hypothesised in the socio-emotional selectivity theory (Carstensen et al., 1999): With higher chronological life, people are thought to be more aware of the limited time left in their life. This increases the motivation to pursue emotionally meaningful goals in life like intimacy or feeling socially embedded in life. According to Carstensen et al. (1999), older couples compared to younger couples are more accepting of their relationship as it is, appreciate what is positive, and ignore what is bothering them. Since a first intervention study focusing on gratitude in older adults showed positive effects (Salces-Cubero et al., 2019), a stronger focus on gratitude for successful aging may be worthwhile, particularly since the older age demographic is often faced with significant challenges like caregiver work for partners or dealing with chronic illness (Pruchno, 2021).

In this age cohort, gratitude for physical affection and/or sexuality was only expressed by some, perhaps surprisingly few, which is unusual given the age independence of these topics (Bucher et al., 2003) and the importance of intimacy in close relationships (Beaulieu et al., 2023). This could be related to the fact that sexuality is not often openly discussed in this particular age cohort due to cultural and societal taboos (Riehl-Emde, 2016) and perhaps even less often in direct contact with an interviewer. Whether the same would be true in younger couples or more anonymous paper-pencil or online questionnaires needs to be addressed in future studies.

Regarding expressions of gratitude, participants mentioned direct expressions, like saying thank you, but also a considerable variety of indirect expressions, like showing affection or small acts of love. Interestingly, even though all participants were grateful for something in their partner, only a third mentioned that they would express their gratitude directly by saying thank you. However, many respondents described indirect expressions of gratitude, like showing appreciative behaviour towards their partner. A variety of behaviours and other indicators were described that the respondents perceived as signs of gratitude. Thus, it became apparent that a variety of subjective ideas of gratitude or forms of gratitude expressions exist that are perceived, interpreted, and recognised as an expression of gratitude by the partner. For example, preparing a favourite dish could be a seen as a way of saying thank you. Some of these indirect expressions of gratitude show parallels to relationship maintenance behaviour described by Gordon and colleagues (2012). They also emphasise the relevance of partner responsiveness to one’s own needs (Reis et al., 2004). A narrow understanding or definition of gratitude expressions in romantic relationships may lead to relevant aspects of relational gratitude being missed.

Indirect expression of gratitude may not always be easily understood by one’s partner. Some interviewees explicitly touched on the difficulty of differentiating what could be recognized as an expression of gratitude as opposed to love, support, or care. Quite frequently, women described that their partners expressed gratitude through acts of love, whereas male respondents rarely mentioned this, neither as their expression nor as an expression of their partner. This lack of congruence between self and partner reports may serve as an indicator that gratitude is expressed and perceived in multi-layered and quite unique ways. Similarly, as Yoshimura and Berzins (2017) state: “The difficulty lies in distinguishing a single message as distinctly one of gratitude rather than a message of affection, compassion, politeness, kindness, or other constructs.” (p. 110). This poses a challenge for the field of gratitude research, since these indirect forms of gratitude expressions are currently not sufficiently covered in self-report questionnaires on gratitude and are more difficult to assess. Additionally, there seems to be a cultural layer in the communication of gratitude. In an intercultural study in Taiwan and the USA, individuals from the USA used bodily contact more while individuals from Taiwan used self-improvement more to show their gratitude (Chang and Algoe, 2020). With the current Western sample in this study, self-improvement was not mentioned, however, physical touch was often mentioned.

Another point worth mentioning here is the notion that within some relationships the role of gratitude was not without ambiguities. For instance, for some participants, expressions of gratitude through gifts were described as “bribery” and one participant completely rejected the notion of gratitude within a relationship, considering it inappropriate, which points to the relevance of context (McNulty and Fincham, 2012). Along this line, Wood et al. (2016) characterized harmful ways of gratitude. According to these authors, gratitude expressions would exist on a continuum from high to low and the perfect expression would be in the *golden mean* (Wood et al., 2016: 142), where gratitude is situationally appropriate and expressed to the right degree, not too much and not too little. This is termed *situation specific optimality* (Ng and Tay, 2020: 317). According to Wood et al. (2016), most authors in psychological research implicitly refer to this virtuous understanding when studying the benefits of gratitude. In the current accounts of study participants, feelings of gratitude seemed to be situationally appropriate and its expression seemed to be within the *golden mean* range.

On a more general level, it is interesting that participants not only mentioned gratitude for the positive actions of their partner, but were also grateful for the partner *not* doing something within the relationship like the partner never having been violent or unfaithful. Potentially, participants evaluated their relationship and compared their partner with prior romantic partners, with other couples from their circle of friends, or with an internalized script of romantic relationships. Through this comparison, gratitude was felt for the positive actions of the partner but also the partner’s refraining from negative behaviours.

The findings of the current study suggest some gender differences. Overall, on a descriptive level, women in this study reported more domains of gratitude and expressions of gratitude than men. One explanation is the traditional Western constructs of masculinity where stoicism and emotional control are central for men (Reeser and Gottzén, 2018). In line with this, men would be less willing or less practised at expressing softer, other-focused emotions such as gratitude (Kashdan et al., 2009). Nevertheless, the male participants reported a considerable number of domains of gratitude within their relationships, which were similar in content to those reported by their female partners, suggesting that we should be cautious about overemphasizing gender differences in this context.

When looking at the content of gratitude expressions, some differences were visible between men and women that may be attributable to the more traditional gender roles potentially prevalent in the older aged sample. Women, for example, mentioned being grateful for their life situation that the partner enabled or being supported in household chores. Additionally, some women, but none of the men, mentioned being grateful that their partner was faithful to them or that the partner refrained from using physical violence in the relationship. It would be interesting to establish whether this finding is an effect of the older age cohort and younger age cohorts would not care to mention the absence of physical violence as a source of gratitude. However, in light of the high prevalence of domestic violence, this may not be likely (Sanz-Barbero et al., 2018). Additionally, men were more likely than women to say that even though they felt gratitude they did not express it. This was mirrored by the women’s expressed experience that they knew their partner was grateful but that he did not express it directly.

### Strengths, limitations and future research

A major strength of the current study is the in-depth analyses of subjective gratitude experiences of couples in long-term relationships, which sheds new light on gratitude in romantic relationships beyond mere self-report questionnaires. However, several limitations need to be addressed. Even though we followed an applied qualitative content analysis in a transparent, rigorous, and rule-oriented fashion, the interpretative coding of interview material remains a subjective process. To address this subjectivity, each step was carefully supervised, and later inter-coder reliability was satisfactory.

Overall, the sample was quite homogenous and consisted of mostly educated heterosexual older couples with high relationship satisfaction. Once again, it seems that couples willing to participate in dyadic research are not representative of the whole population (Barton et al., 2020). Expanding this line of research to samples more heterogeneous regarding relationship satisfaction, age, educational level, sexual orientation, or *non-WEIRD* samples (Henrich et al., 2010) would be worthwhile. By WEIRD we refer to samples from Western, Educated, Industrialized, Rich and Democratic societies (Henrich et al., 2010). For instance, in an intercultural study, individuals from Taiwan communicated their gratitude via self-improvement (Chang and Algoe, 2020) which was not mentioned in the current Western sample.

Another particularity of the study worth mentioning is that data collection took place during restrictions of the COVID-19 pandemic. The outbreak of the pandemic, uncertainty about potential infections and the measures taken to contain the virus, such as restricted freedom of movement or social distancing, profoundly changed everyday life (Heid et al., 2021). If anything, the restrictions of social contacts and experiences of uncertainty may have acted as a magnifier on the relationship and gratitude dynamics within the couples. Many interviewees indeed touched on social restrictions and COVID during the interview.

In future research, it would be interesting to take a closer look at the dynamics of gratitude when it is felt/expressed for specific actions versus on a global level. When one is simply grateful that they have this dear person at their side (non-specific gratitude or general gratitude), then there may be no specific opportunity to express their gratitude even though it may be felt deeply. If, on the other hand, a person has received very specific help, then they can subsequently give thanks in a much more contingent way, i.e. the contingency of thanking is related to the type of behaviour or the area. How are these different patterns of gratitude then related to relationship outcomes? Additionally, it may be worthwhile to formulate a taxonomy of gratitude in close relationships along several dimensions, such as (a) domains of gratitude, (b) specificity of domains vs general feelings of gratitude, and (c) expressed vs felt gratitude.

### Conclusions

The participating older couples shared a large variety of partner and relationship characteristics they were grateful for. For many participants, the experience of gratitude seemed to play an important role in their long-term relationship. For some couples, gratitude was a central part of the couple’s identity, for others, expressions of gratitude were more subtle or indirect. Some even viewed presents as signs of bribery or the concept of gratitude altogether as not applicable within the romantic relationship context. The various forms of indirect expressions of gratitude broaden the current understanding of gratitude in long-term relationships. These often idiosyncratic expressions of gratitude might not be easily identifiable from an outside perspective, potentially leading to an underestimation of the actual levels of gratitude present in the relationship and the potentially beneficial role of gratitude in healthy aging.

## Data Availability

We do not share the data of participants publicly, due to the sensitive nature of the interview data.*
